# Allogeneic Stem Cell Transplantation Combined With Transfusion of Mesenchymal Stem Cells in Primary Myelofibrosis: A Multicenter Retrospective Study

**DOI:** 10.3389/fonc.2021.792142

**Published:** 2022-01-24

**Authors:** Qingyuan Wang, Na Xu, Yu Wang, Xi Zhang, Limin Liu, Huifen Zhou, Hong Wang, Xiang Zhang, Xiaowen Tang, Chengcheng Fu, Miao Miao, Depei Wu

**Affiliations:** ^1^ National Clinical Research Center for Hematologic Diseases, Jiangsu Institute of Hematology, The First Affiliated Hospital of Soochow University, Suzhou, China; ^2^ Institute of Blood and Marrow Transplantation, Collaborative Innovation Center of Hematology, Soochow University, Suzhou, China; ^3^ Key Laboratory of Thrombosis and Hemostasis of Ministry of Health, Suzhou, China; ^4^ Nanfang Hospital, Southern Medical University, Guangzhou, China; ^5^ Peking University People’s Hospital, Peking University Institute of Hematology, National Clinical Research Center for Hematologic Disease, Research Unit of Key Technique for Diagnosis and Treatments of Hematologic Malignancies, Beijing, China; ^6^ Beijing Key Laboratory of Hematopoietic Stem Cell Transplantation, Beijing, China; ^7^ Collaborative Innovation Center of Hematology, Peking University, Beijing, China; ^8^ Xinqiao Hospital, Army Military Medical University, Chongqing, China

**Keywords:** primary myelofibrosis, allogeneic stem cell transplantation, mesenchymal stem cells, graft failure, overall survival

## Abstract

**Background:**

Allogeneic stem cell transplantation (allo-SCT) remains the only effective curative therapy for primary myelofibrosis. Utilization and efficacy of allo-SCT are limited by lethal complications, including engraftment failure, and acute (aGVHD) and chronic graft-versus-host disease (cGVHD). Several clinical trials have explored the use of mesenchymal stem cells (MSCs) in allo-SCT to prevent hematopoietic stem cell (HSC) engraftment failure and control GVHD.

**Methods:**

Clinical data of 17 patients with primary myelofibrosis who underwent allo-SCT combined with *ex vivo* expanded MSC transfusion in four centers from February 2011 to December 2018 were retrospectively analyzed.

**Results:**

All patients received myeloablative conditioning regimen. The median number of transplanted nucleated cells (NCs) per kilogram body weight was 11.18 × 10^8^ (range: 2.63–16.75 × 10^8^), and the median number of CD34^+^ cells was 4.72 × 10^6^ (range: 1.32–8.4 × 10^6^). MSCs were transfused on the day of transplant or on day 7 after transplant. The median MSC infusion number was 6.5 × 10^6^ (range: 0.011–65 × 10^6^). None of the patients experienced primary or secondary graft failure in the study. The median time to neutrophil engraftment was 13 days (range: 11–22 days), and the median time to platelet engraftment was 21 days (range: 12–184 days). The median follow-up time was 40.3 months (range: 1.8–127.8 months). The estimated relapse-free survival (RFS) at 5 years was 79.1%, and overall survival (OS) at 5 years was 64.7%. Analysis showed that the cumulative incidence of aGVHD grade II to IV was 36% (95% CI: 8%–55%) and that of grade III to IV was 26% (95% CI: 0%–45%) at day 100. The cumulative incidence of overall cGVHD at 2 years for the entire study population was 63% (95% CI: 26%–81%). The cumulative incidence of moderate to severe cGVHD at 2 years was 17% (95% CI: 0%–42%). Seven patients died during the study, with 5 patients succumbing from non‐relapse causes and 2 from disease relapse.

**Conclusion:**

The findings of the study indicate that allo-SCT combined with MSC transfusion may represent an effective treatment option for primary myelofibrosis.

## Introduction

Primary myelofibrosis (PMF) is a myeloproliferative neoplasm (MPN) characterized by abnormal proliferation of megakaryocytes, bone marrow (BM) fibrosis, and extramedullary hematopoiesis. Patients with symptomatic PMF have less than 5 years’ median survival (1). Conventional therapies and Janus kinases (JAK) inhibitors are mainly used for palliative purposes and have not been demonstrated to favorably modify disease natural history or prolong patient survival (2). Currently, allogeneic stem cell transplantation (allo-SCT) is the only treatment modality for PMF, and it results in disease remission and restoration of normal hematopoiesis (3).

However, despite significant advances in allo-SCT in MF, it is currently associated with a relatively high rate (range from 24% to 50% at 5 years) of transplant-related deaths or severe morbidity related to graft failure (GF), conditioning regimen-related toxicity, lethal organ injury, infectious complications, and graft-versus-host disease (GVHD) (4–7). Although mortality from conditioning toxicity has been reduced in recent years, studies should explore methods to alleviate GF and GVHD in patients who have undergone allo-SCT. Engraftment and/or graft function can be challenging in patients with PMF undergoing an allo-SCT. GF was reported in 11% of PMF patients that is independently associated with increased mortality in patients who have undergone allo-SCT [hazard ratio (HR): 2.30] (8). In addition, GVHD is another major cause of morbidity and mortality in allo-SCT patients with PMF. The estimated 5-year survival rate of patients who develop GF and grade III–IV aGVHD was 14% and 26%, respectively (8). Therefore, enhancing engraftment as well as decreasing GVHD is a potential strategy for improving efficacy of allo-SCT treatment.

Mesenchymal stem cells (MSCs) are multipotent BM cells capable of regenerating rudimentary bone *in vivo* and supporting hematopoiesis (9). Preclinical animal studies reported improved engraftment of hematopoietic stem cells (HSCs) and decreased risk of GVHD after co-transplantation of MSCs and HSCs (10–12). These results laid a basis for further investigations on clinical application of MSC therapy to improve allo-SCT outcomes in patients with hematological disorders. Currently, MSCs are widely applied in the treatment of hematological disorders. For instance, MSCs have been used in the treatment of aplastic anemia for promoting engraftment. In addition, MSCs are also employed in treating engraftment failure and preventing and alleviating GVHD (13–15). Several clinical studies reported that cotransplantation of autologous or allogeneic MSCs with HSCs promotes BM engraftment (16–18).

To the best of our knowledge, no previous study has explored the outcomes of allo-SCT combined with MSC transfusion for patients with PMF. Therefore, there is a need to evaluate the effect of MSC infusion in combination with allo-SCT in patients with PMF. The current study explored the effects and safety of a single dose of MSC infusion during transplantation procedure in combination with myeloablative conditioning (MAC) regimen as a new allo-SCT modality for patients with PMF attending four independent centers from February 2011 to December 2018.

## Methods

### Eligible Patients

The current study enrolled patients diagnosed with PMF who underwent a first allogeneic BM or peripheral blood stem cell (PBSC) transplant, with MSC transfusion prior to engraftment, admitted at 4 centers between 2011 and 2018. All patients were aged 18–65 years. In addition, patients with PMF progressing to acute leukemia were included in the current study. Patients diagnosed with myelofibrosis originating from polycythemia vera or essential thrombocythemia were excluded from the study. Data on 17 allo-SCTs were retrospectively included in the current study. All patients and donors or their legal guardians provided written informed consent prior to inclusion to the study in accordance with the Declaration of Helsinki.

### Conditioning Regimen and Supportive Care

All patients received a MAC busulfan (BU)/cyclophosphamide (CY)-based regimen. Busulfan 9.6 mg/kg was intravenously administered to patients in 12 doses on days -7 to -5. Sodium valproate was administered for busulfan‐induced seizure prophylaxis. Two doses of CY (3.6 g/m^2^) was intravenously administered on days -4 to -3. Mesna was administered to lower the risk of hemorrhagic cystitis. Granulocyte colony‐stimulating factor (G‐CSF) was administered subcutaneously at a dose of 5–10 μg/kg from day +7 after allogeneic transplantation and was continued until sustained neutrophil engraftment.

Patients with HLA-matched related donors (MRDs) received GVHD prophylaxis comprising cyclosporine A (CsA) and short-term methotrexate (MTX). Notably, one patient received additional antithymocyte globulin (ATG) and mycophenolate mofetil (MMF) owing to old donor age. ATG, CsA, MMF, and MTX were included for GVHD prophylaxis for HLA-matched unrelated donors (MUDs) and haploidentical donors (HIDs). CsA dose was adjusted to 200–300 ng/ml serum levels and was gradually reduced and withdrawn completely on month 9 after transplantation when no GVHD was observed. MTX was administered at 15 mg/m^2^/day on day +1 and at 10 mg/m^2^/day on days +3, +6, and +11 (only for MUDs and HIDs). Here, 15 mg/kg MMF was administered orally twice a day from days -9 to +30 and then gradually decreased by day +40 when no GVHD was observed. ATG was administered on days −5 to −2 at a total dose of 10 mg/kg in haploidentical allo-SCT setting (19).

### Graft Collection and Infusion

Stem cell mobilization of donors was performed by subcutaneous injection of 5 consecutive days of recombinant human G-CSF (rhG-CSF) at a dose of 10 μg/kg/day. The first day of stem cell infusion was denoted as “day 01”, and the second day of infusion was denoted a “day 02”. For MRDs and HIDs, graft source was BM combined with PBSCs. BM was collected on day 01 *via* BM aspiration in the operating room. PBSCs were collected on the following day (day 02) by apheresis using a COBE Spectra device (Gambro BCT, Lakewood, CO, USA). For MUDs, PBSCs were the sole graft source and were collected on day 01. Target nucleated cell (NC) count was expected to achieve 6–10 × 10^8^/kg of recipient weight, minimum 2 × 10^8^/kg under specific occasions. If the target count of cells was insufficient, additional PBSCs were collected on the next day. Fresh BM and PBSCs were infused into the recipient on the day of their collection.

### Definitions and Disease Monitoring

The first day of the absolute neutrophil count (ANC) >0.5 × 10^9^/L for 3 consecutive days was defined as neutrophil engraftment. The first day when the platelet count was >20 × 10^9^/L without transfusion support for 7 consecutive days was defined as platelet engraftment. Primary GF was defined as failure to achieve neutrophil engraftment within the first 28 days after stem cell infusion documentation of autologous reconstitution by chimerism analysis in the absence of relapse. Secondary GF was defined as recurrent ANC ≤0.5 × 10^9^/L after initial engraftment in the absence of reversible causes of drop in counts. Relapse was defined as reappearance or persistence of host cells with pretransplant morphological, cytogenetic, or molecular markers of the disease. Acute GVHD was scored based on a criteria proposed by the 1994 Consensus Conference on Acute GVHD Grading (20). cGVHD was evaluated based on the NIH Consensus Development Project on Criteria for Clinical Trials in Chronic Graft-versus-Host Disease: Diagnosis and Staging Working Report (21). Early mortality referred to death within 60 days after allo-SCT. Polymerase chain reaction was performed to explore donor chimerism weekly from the time of neutrophil recovery. All patients underwent BM examination after allo‐SCT monthly during the first 3 months and every 3–6 months during the following 1–2 years in order to evaluate marrow cellularity and disease status.

### Preparation of Mesenchymal Stem Cells

MSCs were *ex vivo* expanded and derived from donor or relative BM in 11 patients. BM mononuclear cells were separated by Lymphoprep (Axis-Shield, Oslo, Norway) density gradient centrifugation as previously described (15). Washed BM mononuclear cells were resuspended in α modified Eagle’s medium (Gibco, Shanghai, China) supplemented with 10% fetal bovine serum (Gibco, Shanghai, China) and cultured with a density of 1.0 × 10^6^ cells per cm². Cultures were maintained at 37°C in a humidified atmosphere containing 5% CO₂ in 15-cm culture dish (NEST, Wuxi, China). When the cultures were near confluence (>85%), the cells were detached with 0.25% trypsin and EDTA solution (Gibco, Shanghai, China) and cultured at a density of 4,000 cells per cm². When 5 × 10⁶ cells or more were obtained, they were harvested and washed repeatedly. The cells are formulated in saline solution containing 1% human serum albumin. Criteria for release of MSCs for clinical use included spindle-shape cell, no visible cell clumps, absence of contamination by pathogens, viability greater than 92% as determined by trypan blue testing, and immune phenotyping proving expression of CD73, CD29, CD44, CD105, and CD90 surface molecules (>95%) and negative for markers of hematopoietic lineages, CD45, CD14, CD34, and human leukocyte antigen-DR (HLA-DR) receptors (<5%). The complete cell culture process consists of a total of 4 cell passages. All manufacturing activities were performed in strict compliance with National Medical Products Administration’s good manufacturing practice (GMP) standards. In addition, MSCs were obtained from third-party BM source in four patients as previously reported (22). MSCs were obtained from third-party umbilical cord (UC) blood (UCB) and third-party UC sources (iCELL Biotechnology) with the informed consent of the mother in the other 2 patients, as previously reported (23) The immunophenotype of UC-MSCs included positivity for CD13, CD29, CD90, CD44, CD105, and CD73 and negativity for CD14, CD34, CD38, CD45, CD31, and HLA-DR. The immunophenotype of UCB-MSCs was positive for CD29, CD44, CD73, CD90, CD105, and CD166 and negative for CD34, CD45, CD14, and HLA-DR. MSCs were administered as a single dose at the day of stem cell infusion in 5 patients, and the remaining 12 patients received MSCs on day 7 after transplant.

### Statistical Analysis

Baseline characteristics and demographics were recorded. Continuous variables that did not follow normal distribution were expressed as median and range. Time to event duration was calculated in months or days from the date of transplant to the date of the event or last date of follow‐up for patients who were alive throughout the study. Cumulative engraftment rate, cumulative incidence of aGVHD and cGVHD, relapse-free survival (RFS), and overall survival (OS) were explored using Kaplan–Meier estimator. Survival and cumulative incidence estimate at the specified time points also report numbers of patients at risk and two-sided 95% confidence intervals (CIs). Death without aGVHD/cGVHD and relapse was the competing event for estimating GVHD incidence and RFS. Statistical analyses were performed using RStudio (R version 3.6.1).

## Results

### Patient-, Disease-, and Transplantation-Related Characteristics

Characteristics of included patients are presented in **Table 1**. Adult patients (10 males, 7 females) with a median age of 41 years (range: 22–55 years) at diagnosis were included in the current study. All patients were diagnosed with PMF. Notably, 3 patients had leukemic transformation at the time of transplant (patient No. 13, 14, 16). Disease history varied widely different, with diagnosis-to-transplant time ranging from 2 months to 10 years (median: 18 months). Gene mutations were negative in five patients. For driver mutations, a mutation in JAK2 was confirmed in 5 cases, followed by CALR mutation in 3, and none harbored MPL mutation. Six patients harbored ASXL1 gene mutation, and four of them harbored also mutations affecting JAK2 and CALR genes. U2AF1 mutation was found in 3 patients. EZH2 was mutated in 1 and IDH1/2 in 1 who also harbored mutations in K/NRAS. One patient harbored both mutations in SF3B1 and SETBP1. All study patients were categorized according to the DIPSS plus risk category, which was defined by the International Working Group (IWG) for MF (24). Risk profile was intermediate‐1 in 2 patients, intermediate‐2 in 2 patients, and high in 13 patients. Ten patients received ruxolitinib before transplantation, one patient had previously undergone splenectomy. Stable transfusion dependency was observed in 10 patients (5 out of the 10 patients were under ruxolitinib treatment). Comorbidity scores determined using the Hematopoietic Cell Transplantation Comorbidity Index (HCT‐CI) ranged from 0 to 4 (25). Notably, 14 patients were in the 0–1 score group, and 3 patients had HCT‐CI scores from 2 to 4. Thirteen patients were classified as MF‐3 based on the WHO criteria at the time of transplant (26). Severe splenomegaly defined as length of the spleen exceeding the umbilicus was observed in 7 patients. Transplant details are presented in **Table 2**. A total of 5 patients received the graft from MRD, 3 patients received the graft from MUD, and 9 patients received the graft from HID. The graft source was BM-derived stem cells (BMSCs) combined with mobilized PBSCs for all related donors (N = 14). The other 3 patients received mobilized PBSCs from MUDs. Donor and recipient ABO type was compatible in most patients (N = 11). Major incompatibility was observed in 3 patients, minor incompatibility was observed in 2 patients, and bidirectional mismatch was reported in 1 patient. The median donor age was 34 (range: 16–48 years). Median MSC infusion dose count was 6.5 × 10^6^ (range: 0.011–65 × 10^6^).

**Table 1:** Patient- and disease-related characteristics.

Patient No.	Sex	Age, y	Diagnosis	DIPSS‐plus risk	Cytogenetics	Mutations	HCT-CI	ECOG	Transfusion dependency	Time diagnosis-to-alloSCT, m	Splenectomy	Pre-transplant ruxolitinib	Fibrosis grade before allo-SCT	Severe Splenomegaly	Follow up, m
1	F	39	PMF	High	46, XX, del(18)(q12q23)	Negative	3	1	Y	12	N	N	MF-3	N	74.9
2	M	30	PMF	Int-1	Normal	JAK2	1	1	N	12	N	Y	MF-2	N	64.3
3	M	45	PMF	High	48, X, -Y, ?1q-, der(3), t(1;3)(q11;p24), der(4), t(4);?(p15);?[2]/46, XY[8]	CALR	4	2	Y	120	N	Y	MF-3	N	56.8
4	M	47	PMF	High	47, idem, +21	JAK2, ASXL1	3	2	Y	4	N	Y	MF-2	Y	50.5
5	M	35	PMF	Int-1	Normal	CALR	0	1	N	12	N	Y	MF-3	Y	2.1
6	F	49	PMF	Int-2	Normal	Negative	1	1	Y	36	N	N	MF-2	N	11.6
7	M	52	PMF	High	48, XY, +8	JAK2, ASXL1	0	1	N	42	N	Y	MF-3	N	6.5
8	F	25	PMF	Int-2	Normal	Negative	0	1	N	24	N	Y	MF-3	Y	31.7
9	M	42	PMF	High	Normal	ASXL1	1	1	Y	2	N	N	MF-3	N	71.3
10	F	31	PMF	High	47, XX, +8	U2AF1, SETD2	0	2	Y	2	N	N	MF-3	N	54.7
11	M	25	PMF	High	47, XY, +8	U2AF1, ASXL1	0	2	N	24	N	N	MF-2	Y	1.8
12	F	51	PMF	High	Normal	Negative	1	2	Y	36	N	N	MF-3	N	77.4
13	M	41	PMF	High	45, XY, -9, +10, -7, 5q-	IDH1, IDH2, NRAS, JAK2, KRAS, U2AF1, ASXL1	0	0	Y	18	Y	Y	MF-3	Y	32.9
14	M	51	PMF	High	47, XY, +8, inv(9)(p12q13)	RUNX1, CALR, EZH2, ASXL1	0	0	N	42	N	Y	MF-3	N	19.7
15	F	41	PMF	High	46, XX, del(20)(q11)	SF3B1, SETBP1	0	0	Y	11	N	Y	MF-3	Y	40.3
16	F	55	PMF	High	45, XX, t(3,3)(q21:q26), -7	JAK2-V617F, EVI1, TET2, HOX11	1	1	Y	8.7	N	Y	MF-3	Y	1.8
17	M	22	PMF	High	Normal	Negative	0	2	N	48	N	N	MF-3	N	127.8

F, female; M, male; PMF, primary myelofibrosis; Int-1, intermediate‐1; Int-2, intermediate‐2; HCT-CI, hematopoietic cell transplantation-comorbidity index; ECOG, Eastern Cooperative Oncology Group score; Y, yes; N, no.

**Table 2:** Transplantation-related characteristics.

Patient No.	GVHD prophylaxis	Donor type	Donor/recipient ABO type	Donor age	Donor sex	NCs dose count, ×10^8^/kg	CD34^+^ cell dose count, ×10^6^/kg	MSCs dose count, ×10^6^	Time to neutrophil engraftment	Time to platelet engraftment	aGVHD grade	cGVHD grade	Complications	Current status	Cause of death
1	CsA+MTX	MRD	AB+/A+	42	F	11.3	5.23	6.5	11	15	/	Mild	/	Alive	/
2	ATG+MMF+CsA+MTX	MUD	A+/A+	45	F	2.63	5	6.5	13	17	/	/	/	Alive	/
3	ATG+MMF+CsA+MTX	HID	A+/A+	26	M	12.72	6.31	6.7	17	21	I	Mild	/	Alive	/
4	ATG+MMF+CsA+MTX	HID	A+/O+	26	M	4.94	7.8	6.5	20	63	III	/	/	Alive	/
5	ATG+MMF+CsA+MTX	HID	O+/O+	34	F	16.67	4.84	6.5	13	26	/	/	TMA	Died on day 64	TMA
6	ATG+MMF+CsA+MTX	HID	A+/A+	45	M	16.75	5.36	6	12	15	IV	Mild	Heart failure, cystitis	Died on day 349	Heart failure
7	ATG+MMF+CsA+MTX	HID	O+/O+	20	M	14.44	4.47	6.5	12	18	II	Mild	Pneumonia	Died on day 195	Toxic epidermal necrolysis syndrome
8	ATG+MMF+CsA+MTX	MUD	O+/A+	24	F	6.72	4.5	6	11	60	/	/	CMV/EBV pneumonia	Alive	/
9	CsA+MTX	MRD	A+/A+	48	F	12.27	4.2	6.5	11	12	I	Mild	/	Died on day 2138	Relapse
10	CsA+MTX	MRD	O+/O+	30	F	15.03	4.59	60	13	30	/	Mild	/	Alive	/
11	ATG+MMF+CsA+MTX	HID	AB+/B+	47	M	11.18	5.42	65	11	17	II	/	Immunologic cerebral vasculitis, pneumonia	Died on day 53	Immunologic cerebral vasculitis
12	ATG+MMF+CsA+MTX	MUD	A+/B+	20	M	6.4	8.4	50	22	184	/	Moderate	/	Alive	/
13	ATG+MMF+CsA+MTX	HID	O+/B+	16	M	10.69	3.18	0.011	11	13	III	/	/	Alive	/
14	ATG+MMF+CsA+MTX	MRD	O+/O+	42	F	10.47	3.56	0.011	12	19	/	Mild	/	Died on day 590	Relapse
15	CsA+MTX	MRD	O+/O+	35	F	10.47	1.72	0.011	13	32	III	Mild	/	Alive	/
16	ATG+MMF+CsA+MTX	HID	B+/B+	27	F	6.58	1.32	0.011	18	37	/	/	CMV reactivation, cystitis	Died on day 55	CMV pneumonia
17	ATG+MMF+CsA+MTX	HID	A+/A+	46	M	11.4	/	60	16	103	/	/	Pneumonia, drug-induced liver injury	Alive	/

GVHD, graft-versus-host disease; CsA, cyclosporine A; ATG, antithymocyte immunoglobulin; MMF, mycophenolate mofetil; MTX, methotrexate; F, female; M, male; MRD, matched related donor; MUD, matched unrelated donor; HID, haploidentical donor; NCs, nucleated cells; MSCs, mesenchymal stem cells; CMV, cytomegalovirus; EBV, Epstein–Barr virus; “/”, not occurred; TMA, thrombotic microangiopathy.

### Engraftment

**Figure 1:**
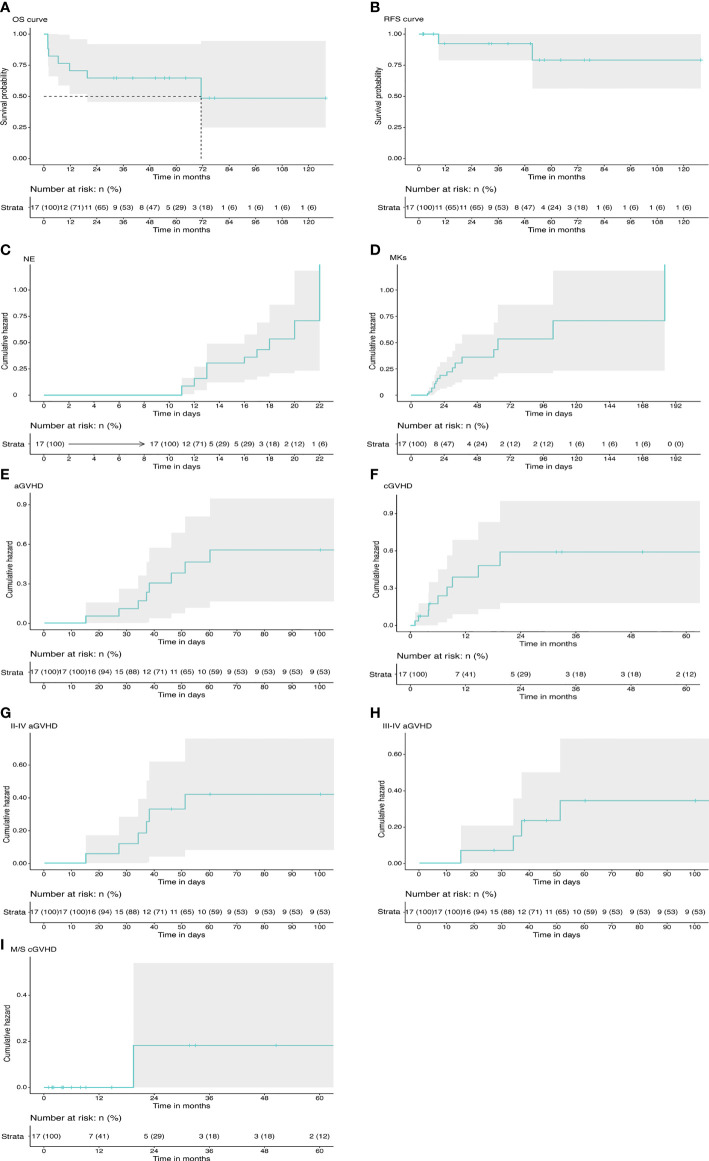
Transplant outcomes in primary myelofibrosis. **(A)** The probability of OS. **(B)** The probability of RFS. **(C)** The cumulative incidence of neutrophil engraftment. **(D)** The cumulative incidence of platelet engraftment. **(E)** The total cumulative incidence of aGVHD. **(F)** The total cumulative incidence of cGVHD. **(G)** The cumulative incidence of grade II–IV aGVHD. **(H)** The cumulative incidence of grade III–IV aGVHD. **(I)** The cumulative incidence of moderate to severe cGVHD. OS, overall survival; RFS, relapse-free survival; NE, neutrophil; MK, megakaryocyte; aCVHD, acute graft-versus-host disease; cGVHD, chronic graft-versus-host disease.

Median number of transplanted NCs per kilogram body weight was 11.18 × 10^8^ (range: 2.63–16.75 × 10^8^) and that of CD34^+^ cells was 4.72 × 10^6^ (range: 1.32–8.4 ×10^6^) (CD34^+^ proportion was not detected in one of the patients) (**Table 2**). Notably, no cases of primary or secondary GF were observed in the study. All patients achieved sustained full donor chimerism in the peripheral blood except 2 relapse patients. The findings showed that the engraftment rate was 100% with a median time to neutrophil engraftment of 13 days (range: 11–22 days). The cumulative neutrophil engraftment rate was 71% (95% CI: 39%–86%) at day +15 and 100% at day +28. Cumulative probability of platelet engraftment was 59% (95% CI: 27%–77%) at day +28 and 88% (95% CI: 57%–97%) at day +100 (**Figures 1C, D**). Platelet recovery showed a median time of 21 days (range: 12–184 days). All patients presented sustained engraftment except for the two relapse patients.

### Graft-Versus-Host Disease

Details on GVHD are presented in **Table 2** and **Figures 1E–I**. Acute GVHD was observed in 8 patients, with all cases presenting in the first 100 days after transplantation. Four patients presented with grade III–IV aGVHD. Notably, 3 out of the 4 patients had received graft from an haploidentical related donor. Cumulative incidence of grade I–IV aGVHD was 47% (95% CI: 17%–66%). Incidence of acute GVHD grade II–IV was 36% (95% CI: 8%–55%) and incidence of grade III–IV was 26% (95% CI: 0%–45%) at day 100.

cGVHD was observed in 9 patients, with onset occurring in the first 2 years after transplantation for all cases. cGVHD was mild in 8 cases and moderate in 1 case who received the graft from a MUD. No severe cGVHD was observed. Cumulative incidence of overall cGVHD at 2 years for the entire study population was 63% (95% CI: 26%–81%). Cumulative incidence of moderate to severe cGVHD at 2 years was 17% (95% CI: 0%–42%). All patients had clinical response to immunosuppressive therapy, and no deaths secondary to cGVHD was reported.

### Transplant-Related Complications

A total of 7 patients died during follow‐up (median: 40.3 months). The death of 5 out of the 7 patients was attributed to non‐relapse causes (patient nos. 5, 6, 7, 11, and 16), and 2 out of the 5 patients experienced early mortality (1.8 and 1.8 months). Causes of early death were immunologic cerebral vasculitis and cytomegalovirus (CMV) pneumonia. Notably, the patient who succumbed to immunologic cerebral vasculitis had been diagnosed with congenital abnormalities of cerebrovascular maldevelopment and had a long history of recurrent epilepsy before transplant (patient no. 11). Patient no. 6 died at 11.6 months after transplant due to heart failure that was caused by persistent atrial fibrillation onset before PMF diagnosis. Patient no. 7 died from toxic epidermal necrolysis syndrome at 6.5 months. Two patients (12%) presented with CMV reactivation, and both developed CMV pneumonia.

Safety profile of the patients was monitored throughout the study. The findings showed that no patients presented with allergic hypersensitivity, fever, hypertension, pulmonary embolism, or other serious complications directly related to MSC infusion.

### Relapse and Survival Rates

Disease relapse was observed in 2 cases at 51.4 and 9 months after transplantation (patient no. 9 and patient no. 14, respectively). The two patients harbored mutations in ASXL1 gene and received stem cells from MRD. The patients died from disease progression without undergoing a second transplant. Outcome information of patients is presented in **Table 2** and **Figures 1A, B**. Estimated RFS at 5 years was 79% (95% CI: 56%–100%). Patients estimated that OS at 5 years was 65% (95% CI: 46%–92%). Median follow‐up duration was 40.3 months (range: 1.8–127.8 months).

## Discussion

Allo-SCT is the only treatment modality for curing PMF. Despite the benefit, the associated comorbidities coupled with relatively poor engraftment limit extensive utilization of allo-SCT even in symptomatic PMF patients (4, 27). Thus, a supplementary remedy to boost BM engraftment should be developed to enhance clinical outcomes after allo-SCT. The current study explored whether MSC transfusion can improve transplantation outcomes (engraftment, GVHD, and relapse) in an allo-SCT setting.

GF continues to remain a major barrier of allo-HCT in MF patients, and rates varying from 2% to as high as 28% have been reported in different settings (6, 27). Coupled with GF, delayed hematopoietic reconstitution was also observed. CIBMTR data previously reported a cumulative incidence of neutrophil engraftment of 84% at 28 days and 97% at 100 days. The probability of platelet engraftment was significantly lower with 47% at 28 days and 77% at 100 days (5). In the current study, all patients achieved successful engraftment and faster hematopoietic reconstitution with 100% neutrophil engraftment at 28 days and 59% platelet engraftment incidence at 28 days and 88% at 100 days. Addition of MSC grafts may be beneficial to improve engraftment. This finding aligned with that reported by Liu et al. (28) that MSC coinfusion improved platelet recovery. In another pediatric study, Ball et al. (17) reported similar findings that cotransplanted donor BM-derived MSCs with HSCs resulted in sustained hematopoietic engraftment. Studies report that infusion of MSCs is effective in the treatment of many diseases such as ischemic stroke, diabetes mellitus type I, and hematological diseases owing to their immunomodulatory, angiogenic, antiapoptotic, and antifibrotic therapeutic activities and their ability to support stem cells (29, 30). In addition, MSCs have been utilized as an adjuvant cellular therapy to promote rapid hematopoietic reconstitution in allo-SCT patients by inhibiting apoptosis and differentiation of HSCs and inducing engraftment of precursor cells in the BM niche (31, 32). Mounting evidence has identified that most clinical effects exhibited by MSCs are mainly linked to their paracrine effects (33). MSCs produce factors such as CXCL12 stem cell factor (SCF) that can help in recruiting HSCs and supporting hematopoiesis (34). MSC conditioned medium administration also seemed to enhance BM engraftment in part by restoring vasculature *via* pleiotrophin production (35). Moreover, the type of conditioning (RIC vs. MAC or non-MAC) might also affect the occurrence of GF (27). A reduced-intensity conditioning (RIC) trial comparing fludarabine in combination with busulfan or thiotepa with MF patients confirmed a GF rate of 14% vs. 10% (36). Relatively high engraftment in the current study can be partly attributed to MAC regimen with busulfan that lowers the risk of rejection (37).

GVHD represents another major challenge that results in life-threatening complications after allo-SCT in PMF patients. Although unidentified, in theory, the inflammatory cytokines that mediate aGVHD are also implicated in mediation of the constitutional symptoms in PMF (38). Moreover, a relatively higher number of transfused cells may also contribute to a higher incidence of GVHD in PMF patients. By inhibiting the activation and proliferation of activated T cells, limiting tissue damage, and promoting tissue regeneration, application of MSCs in aGVHD treatment has achieved great success (39, 40). Distinguished from the treatment of GVHD, whether infusion of MSCs can prevent GVHD remains inconclusive (32, 41). In our study, MSCs did not show a significant effect on the incidence of GVHD, as indicated by the incidence of 36% in grade II–IV aGVHD and 63% incidence of cGVHD by 2 years. This finding is consistent with results from Liu et al. (28) and Lee et al. (42), indicating that coinfusion of MSC had no effect on GVHD incidence. Similarly, the incidence of GVHD in the current study is comparable to that of a non-MSC-based allo-SCT trial with MF patients (6). This result may be attributed to the relatively lower dose and single infusion of MSCs compared with a previous report that indicated that MSCs preferably be administered at an average dose of 1.0 × 10^6^/kg (43). The observed slightly increased incidence of GVHD in the entire group can be attributed to the low cases of MRD transplant.

Some studies reported a higher incidence of CMV/EBV reactivation and disease recurrence owing to immuno-suppressive effects of MSCs (44, 45). Nevertheless, it is still a topic of debate. Other studies report that MSC transfusion does not increase the incidence of infection or disease recurrence during transplant or for aGVHD treatment (17, 43, 46). In the current study, neither the reactivation of CMV/EBV nor disease relapse was worsened by MSC transfusion, which was in line with previous studies. This may partly be due to the *in vivo* activity of MSCs that lasts 2 weeks with no significant effect on long-term infection and disease relapse. Out of the 5 patients who died from non-relapse reasons, 3 presented with severe splenomegaly and grade 3 fibrosis, and all 5 patients received graft from an HID. A study based on data from the European Society for Blood and Marrow Transplantation (EBMT) recruiting 103 MF allo-SCT patients reported that HLA-mismatch is a major risk factor for transplant-related mortality (6). A previous study recruiting mostly MAC transplants (83%) reported a 5-year OS of 30%–40% depending on donor sources (4). The 5-year OS was reported as 53.0% in the MAC cohort according to a large EBMT study (7). The estimated survival rates in the current study for RFS and OS at 5 years were 79.1% and 64.7% with a median follow-up of 40.3 months. The relatively higher OS can be partly attributed to the younger median age (41 years) of patients involved in the current study. Moreover, the higher survival rate compared with that of high-risk and young patients with MF who did not undergo allo-SCT corroborated potential curative effect of allo-SCT for PMF patients even with leukemia transformation (47).

This is the first report on allo-SCT combined with MSC transfusion in patients affecting PMF. Despite lack of a control arm, the present results showed promising amelioration of engraftment and increase in survival. These findings indicate that allo-SCT combined with MSC transfusion might be an attractive approach for treatment of PMF patients. Limitations of our study are those inherent to multiple center-based retrospective analyses including a lack of clarity for physician choice of time and dose of MSC infusion and heterogeneous data with GVHD prophylaxis and therapies associated with supportive care. MSC dose varied greatly in previous literature (41). Although it was reported that MSCs exerted an effect in a dose-dependent manner regarding engraftment promotion (48), the impact of MSC dose on engraftment still remains to be determined. Whereas GVHD prevention was reported not correlated with MSC dose (45). Concordantly, optimal timing of MSC infusion is inconclusive and may depend on the infusion purpose (17, 43, 49). Further prospective, controlled, large-scale studies should be conducted to validate the finding obtained in the current study.

## Data Availability Statement

The original contributions presented in the study are included in the article/supplementary material. Further inquiries can be directed to the corresponding authors.

## Ethics Statement

The studies involving human participants were reviewed and approved by the ethics committee of all the four centers involved in the study. The patients/participants provided their written informed consent to participate in this study.

## Author Contributions

QW designed the research study, collected the data, analyzed the data, and wrote the article. NX designed the research study, collected the data, and analyzed the data. YW and XiZ analyzed the data. LL contributed to the data analysis and article writing. HZ, HW, and XiaZ contributed to the data analysis. XT and CF contributed to the external validation. MM and DW contributed to the research design, data analysis, writing the article, and supervision of the study. All authors read and approved the final article.

## Funding

This study was supported by the National Natural Science Foundation of China (81730003), National Science and Technology Major Project (2017ZX09304021), National Key R&D Program of China (2019YFC0840604, 2017YFA0104502), Key R&D Program of Jiangsu Province (BE2019798), Priority Academic Program Development of Jiangsu Higher Education Institutions (PAPD), Jiangsu Medical Outstanding Talents Project (JCRCA2016002), Jiangsu Provincial Key Medical Center (YXZXA2016002), and Suzhou Science and Technology Program Project (SLT201911).

## Conflict of Interest

The authors declare that the research was conducted in the absence of any commercial or financial relationships that could be construed as a potential conflict of interest.

## Publisher’s Note

All claims expressed in this article are solely those of the authors and do not necessarily represent those of their affiliated organizations, or those of the publisher, the editors and the reviewers. Any product that may be evaluated in this article, or claim that may be made by its manufacturer, is not guaranteed or endorsed by the publisher.
